# Bis(2-amino-6-methyl­pyrimidin-1-ium-4-olate-κ^2^ *N* ^3^,*O*)bis­(nitrato-κ^2^ *O*,*O*′)cadmium(II)

**DOI:** 10.1107/S1600536810015874

**Published:** 2010-05-08

**Authors:** Kamel Kaabi, Meher El Glaoui, P. S. Pereira Silva, M. Ramos Silva, Cherif Ben Nasr

**Affiliations:** a Laboratoire de Chimie des Matériaux, Faculté des Sciences de Bizerte, 7021 Zarzouna, Tunisia; b CEMDRX, Physics Department, University of Coimbra, P-3004-516 Coimbra, Portugal

## Abstract

In the title compound, [Cd(NO_3_)_2_(C_5_H_7_N_3_O)_2_], the Cd^II^ atom is eight-coordinated by two amine N atoms and two O atoms from two zwitterionic, biodentate 2-amino-6-methyl­pyrimidin-1-ium-4-olate ligands and by four O atoms from two nitrate groups. Intra­molecular N—H⋯O hydrogen bonds occur. The crystal packing is stabilized by inter­molecular N—H⋯O and C—H⋯O hydrogen bonds, two of which are bifurcated, between the nitrate anions and the organic groups.

## Related literature

For common applications of this material, see: Aminabhavi *et al.* (1986 ▶); Ye *et al.* (2008 ▶). For the geometry around the Cd atom, see: Han *et al.* (2008 ▶).
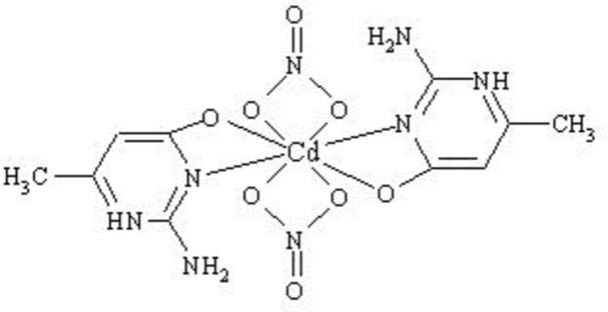


## Experimental

### 

#### Crystal data

[Cd(NO_3_)_2_(C_5_H_7_N_3_O)_2_]
*M* *_r_* = 486.69
Triclinic, 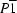

*a* = 7.7230 (12) Å
*b* = 9.5247 (16) Å
*c* = 13.113 (2) Å
α = 70.198 (9)°
β = 81.954 (8)°
γ = 69.840 (8)°
*V* = 851.7 (2) Å^3^
*Z* = 2
Mo *K*α radiation
μ = 1.34 mm^−1^
*T* = 293 K
0.46 × 0.26 × 0.12 mm

#### Data collection

Bruker APEXII CCD area-detector diffractometer
Absorption correction: multi-scan (*SADABS*; Sheldrick, 2003 ▶) *T*_min_ = 0.455, *T*_max_ = 0.851
17615 measured reflections
4031 independent reflections
3603 reflections with *I* > 2σ(*I*)
*R*_int_ = 0.072

#### Refinement

*R*[*F* ^2^ > 2σ(*F* ^2^)] = 0.035
*wR*(*F* ^2^) = 0.084
*S* = 1.21
4031 reflections
244 parameters
40 restraints
H-atom parameters constrained
Δρ_max_ = 0.90 e Å^−3^
Δρ_min_ = −0.53 e Å^−3^

### 

Data collection: *APEX2* (Bruker, 2003 ▶); cell refinement: *SAINT* (Bruker, 2003 ▶); data reduction: *SAINT*; program(s) used to solve structure: *SHELXS97* (Sheldrick, 2008 ▶); program(s) used to refine structure: *SHELXL97* (Sheldrick, 2008 ▶); molecular graphics: *PLATON* (Spek, 2009 ▶); software used to prepare material for publication: *SHELXL97*.

## Supplementary Material

Crystal structure: contains datablocks global, I. DOI: 10.1107/S1600536810015874/bg2342sup1.cif

Structure factors: contains datablocks I. DOI: 10.1107/S1600536810015874/bg2342Isup2.hkl

Additional supplementary materials:  crystallographic information; 3D view; checkCIF report

## Figures and Tables

**Table 1:** Hydrogen-bond geometry (Å, °)

*D*—H⋯*A*	*D*—H	H⋯*A*	*D*⋯*A*	*D*—H⋯*A*
N5—H5⋯O2*A*^i^	0.86	1.97	2.779 (3)	156
N6—H6*A*⋯O7	0.86	2.26	3.090 (6)	163
N6—H6*B*⋯O4^ii^	0.86	2.34	2.892 (4)	122
N6—H6*B*⋯O2*A*^i^	0.86	2.56	3.230 (5)	135
N5*A*—H5*A*⋯O2^iii^	0.86	2.17	2.935 (3)	149
N6*A*—H6*C*⋯O5	0.86	2.36	3.169 (4)	157
N6*A*—H6*D*⋯O2^iii^	0.86	2.25	2.996 (4)	145
N6*A*—H6*D*⋯O3^iv^	0.86	2.26	2.765 (4)	117
C3—H3⋯O6^v^	0.93	2.31	3.160 (5)	152
C3*A*—H3*A*⋯O8^vi^	0.93	2.42	3.301 (5)	157

Symmetry codes: (i) 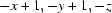; (ii) 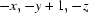; (iii) 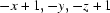; (iv) 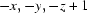; (v) 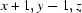; (vi) 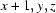.

**Fig. 1.**
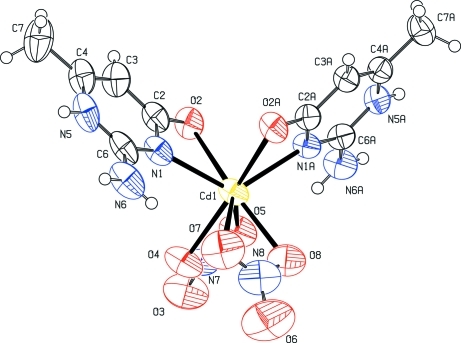
A view of (I), showing 50% probability displacement ellipsoids and arbitrary spheres for the H atoms.

**Fig. 2.**
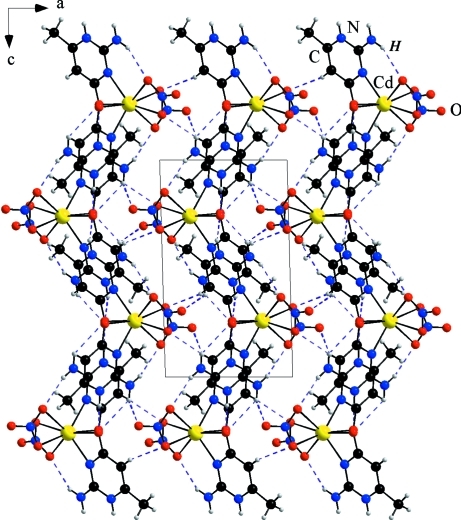
The packing diagram of the compound viewed down the b axis. Hydrogen bonds are shown as dashed lines.

## supplementary crystallographic information

### Comment

Chemists and physicists of the solid state have shown an increasing interest in the study of the metal-organic coordination compounds in recent years owing to their interesting properties such as fluorescence and dielectric behaviour. (Aminabhavi *et al.*, 1986; Ye *et al.*, 2008).

Here we report the synthesis and crystal structure of the title compound Cd(NO_3_)_2_(C_5_H_7_N_3_O)_2_. In the atomic arrangement of this material, the distorted polyhedral Cd environment contains two nitrate anions and two organic moieties. Each one of them is coordinated in the bidental mode; the Cd^II^ is thus eight coordinated (Fig.1), with normal bond distances and angles around the cation (Han *et al.*, 2008). The polyhedra are interconnected by a set of N—H···O and C—H···O hydrogen bonds generated by the organic entities (Table 1). Two of the H-bonds present, N6—H6B···(O2A, O4) and N6A—H6D···(O2, O3), are bifurcated. Fig. 2 shows the results of H-bonding, in the form of undulated columns running along the c axis, laterally interlinked to form a three dimensional infinite network

### Experimental

A solution of Cd(NO_3_)_2_ (0.1 mmol) in ethanol was added dropwise to a solution of 2-Amino-4-hydroxy-6-methylpyrimidine 0.1 mmol in ethanol. After stirring for 30 min, the mixture was filtered. Crystals suitable for X-ray analysis were obtained by evaporating the filtrate at room temperature.

### Refinement

All H atoms were located in a difference Fourier synthesis, placed in calculated positions and refined as riding on their parent atoms, using SHELXL97 (Sheldrick, 2008) defaults (N-H: 0.86Å, C-H: 0.93-0.97Å; *U*_iso_(H) = 1.2*U*_eq_(N,C)). The original structure validation reported that the U values of the coordinating oxygen atoms were too large in relation to the Cd centre, causing the Hirshfeld test to fail. This may be due to mild disorder of the nitrate anions. Restraints on the anisotropic temperature factors (DELU and SIMU instructions in SHELXL97, Sheldrick, 2008) were used to obtain more meaningful anisotropic displacement ellipsoids.

### Crystal data

**Table:**

[Cd(NO_3_)_2_(C_5_H_7_N_3_O)_2_]	*Z* = 2
*M**_r_* = 486.69	*F*(000) = 484
Triclinic, *P*1	*D*_x_ = 1.898 Mg m^−^^3^
Hall symbol: -P 1	Mo *K*α radiation, λ = 0.71073 Å
*a* = 7.7230 (12) Å	Cell parameters from 5520 reflections
*b* = 9.5247 (16) Å	θ = 2.4–28.1°
*c* = 13.113 (2) Å	µ = 1.34 mm^−^^1^
α = 70.198 (9)°	*T* = 293 K
β = 81.954 (8)°	Prism, pale yellow
γ = 69.840 (8)°	0.46 × 0.26 × 0.12 mm
*V* = 851.7 (2) Å^3^	

### Data collection

**Table:**

Bruker APEXII CCD area-detector diffractometer	4031 independent reflections
Radiation source: fine-focus sealed tube	3603 reflections with *I* > 2σ(*I*)
graphite	*R*_int_ = 0.072
φ and ω scans	θ_max_ = 28.1°, θ_min_ = 1.7°
Absorption correction: multi-scan (*SADABS*; Sheldrick, 2003)	*h* = −10→10
*T*_min_ = 0.455, *T*_max_ = 0.851	*k* = −12→12
17615 measured reflections	*l* = −17→17

### Refinement

**Table:**

Refinement on *F*^2^	Primary atom site location: structure-invariant direct methods
Least-squares matrix: full	Secondary atom site location: difference Fourier map
*R*[*F*^2^ > 2σ(*F*^2^)] = 0.035	Hydrogen site location: inferred from neighbouring sites
*wR*(*F*^2^) = 0.084	H-atom parameters constrained
*S* = 1.21	*w* = 1/[σ^2^(*F*_o_^2^) + (0.0212*P*)^2^ + 0.4728*P*] where *P* = (*F*_o_^2^ + 2*F*_c_^2^)/3
4031 reflections	(Δ/σ)_max_ = 0.001
244 parameters	Δρ_max_ = 0.90 e Å^−^^3^
40 restraints	Δρ_min_ = −0.53 e Å^−^^3^

### Special details

**Table:**

Geometry. All esds (except the esd in the dihedral angle between two l.s. planes) are estimated using the full covariance matrix. The cell esds are taken into account individually in the estimation of esds in distances, angles and torsion angles; correlations between esds in cell parameters are only used when they are defined by crystal symmetry. An approximate (isotropic) treatment of cell esds is used for estimating esds involving l.s. planes.
Refinement. Refinement of *F*^2^ against ALL reflections. The weighted *R*-factor wR and goodness of fit *S* are based on *F*^2^, conventional *R*-factors *R* are based on *F*, with *F* set to zero for negative *F*^2^. The threshold expression of *F*^2^ > σ(*F*^2^) is used only for calculating *R*-factors(gt) etc. and is not relevant to the choice of reflections for refinement. *R*-factors based on *F*^2^ are statistically about twice as large as those based on *F*, and *R*- factors based on ALL data will be even larger.

### Fractional atomic coordinates and isotropic or equivalent isotropic displacement parameters (Å^2^)

**Table:**

	*x*	*y*	*z*	*U*_iso_*/*U*_eq_	
Cd1	0.22645 (3)	0.32956 (3)	0.258827 (17)	0.04945 (10)	
O2	0.4649 (4)	0.0693 (3)	0.24489 (17)	0.0565 (6)	
N1	0.3762 (4)	0.2872 (3)	0.1050 (2)	0.0498 (7)	
N5	0.5178 (5)	0.3015 (4)	−0.0651 (2)	0.0639 (9)	
H5	0.5236	0.3549	−0.1324	0.077*	
N6	0.2800 (6)	0.5130 (4)	−0.0387 (3)	0.0745 (11)	
H6A	0.1992	0.5558	0.0033	0.089*	
H6B	0.2888	0.5650	−0.1060	0.089*	
C2	0.4911 (5)	0.1373 (4)	0.1459 (2)	0.0507 (8)	
C3	0.6252 (6)	0.0693 (4)	0.0768 (3)	0.0601 (10)	
H3	0.7046	−0.0329	0.1039	0.072*	
C4	0.6385 (6)	0.1518 (5)	−0.0279 (3)	0.0623 (10)	
C6	0.3913 (6)	0.3665 (4)	0.0005 (3)	0.0556 (9)	
C7	0.7740 (9)	0.0931 (6)	−0.1087 (3)	0.0917 (17)	
H7A	0.8576	−0.0085	−0.0729	0.138*	
H7B	0.8420	0.1653	−0.1425	0.138*	
H7C	0.7097	0.0849	−0.1629	0.138*	
O2A	0.4636 (4)	0.4609 (3)	0.25671 (16)	0.0533 (6)	
N1A	0.3893 (4)	0.2887 (3)	0.40365 (19)	0.0438 (6)	
N5A	0.5494 (4)	0.1982 (3)	0.56308 (19)	0.0488 (7)	
H5A	0.5655	0.1384	0.6293	0.059*	
N6A	0.3128 (5)	0.1088 (4)	0.5545 (2)	0.0619 (8)	
H6C	0.2276	0.1078	0.5192	0.074*	
H6D	0.3304	0.0510	0.6211	0.074*	
C2A	0.4959 (5)	0.3809 (3)	0.3549 (2)	0.0423 (7)	
C3A	0.6342 (5)	0.3833 (4)	0.4149 (2)	0.0456 (7)	
H3A	0.7059	0.4487	0.3831	0.055*	
C4A	0.6597 (5)	0.2896 (4)	0.5181 (2)	0.0453 (7)	
C6A	0.4159 (5)	0.1988 (3)	0.5068 (2)	0.0443 (7)	
C7A	0.8029 (6)	0.2786 (5)	0.5881 (3)	0.0638 (11)	
H7D	0.8735	0.3460	0.5474	0.096*	
H7E	0.8832	0.1719	0.6124	0.096*	
H7F	0.7443	0.3110	0.6497	0.096*	
O3	−0.1415 (5)	0.0957 (4)	0.3034 (3)	0.0956 (10)	
O4	−0.0274 (4)	0.2774 (3)	0.2105 (2)	0.0698 (7)	
O5	0.0767 (4)	0.1314 (3)	0.3670 (2)	0.0715 (7)	
N7	−0.0335 (4)	0.1660 (3)	0.2942 (3)	0.0566 (7)	
O6	−0.2209 (6)	0.7168 (5)	0.2270 (4)	0.1398 (18)	
O7	0.0306 (6)	0.6029 (4)	0.1502 (3)	0.0970 (10)	
O8	−0.0144 (4)	0.5168 (3)	0.3197 (3)	0.0777 (8)	
N8	−0.0712 (7)	0.6164 (4)	0.2318 (4)	0.0848 (12)	

### Atomic displacement parameters (Å^2^)

**Table:**

	*U*^11^	*U*^22^	*U*^33^	*U*^12^	*U*^13^	*U*^23^
Cd1	0.05007 (18)	0.04931 (15)	0.03951 (15)	−0.02325 (12)	−0.01013 (11)	0.00889 (11)
O2	0.0728 (18)	0.0538 (13)	0.0279 (10)	−0.0242 (12)	−0.0036 (10)	0.0106 (9)
N1	0.0603 (18)	0.0503 (14)	0.0319 (12)	−0.0293 (13)	−0.0118 (12)	0.0116 (11)
N5	0.106 (3)	0.0681 (19)	0.0242 (12)	−0.056 (2)	−0.0100 (15)	0.0095 (13)
N6	0.098 (3)	0.0601 (18)	0.0487 (17)	−0.0347 (18)	−0.0312 (18)	0.0240 (14)
C2	0.064 (2)	0.0525 (18)	0.0323 (15)	−0.0304 (16)	−0.0103 (14)	0.0062 (13)
C3	0.084 (3)	0.0541 (19)	0.0358 (16)	−0.0302 (19)	0.0002 (17)	0.0016 (15)
C4	0.094 (3)	0.067 (2)	0.0363 (16)	−0.049 (2)	0.0020 (18)	−0.0056 (16)
C6	0.077 (3)	0.0561 (19)	0.0347 (16)	−0.0423 (18)	−0.0224 (17)	0.0137 (14)
C7	0.142 (5)	0.100 (3)	0.049 (2)	−0.070 (4)	0.027 (3)	−0.023 (2)
O2A	0.0667 (17)	0.0557 (13)	0.0286 (10)	−0.0307 (12)	−0.0075 (10)	0.0115 (9)
N1A	0.0449 (15)	0.0420 (13)	0.0320 (12)	−0.0162 (11)	−0.0046 (10)	0.0076 (10)
N5A	0.0604 (18)	0.0455 (14)	0.0263 (11)	−0.0142 (13)	−0.0079 (11)	0.0063 (10)
N6A	0.064 (2)	0.0629 (18)	0.0420 (15)	−0.0308 (16)	−0.0009 (14)	0.0144 (13)
C2A	0.0467 (18)	0.0402 (15)	0.0298 (13)	−0.0139 (13)	−0.0013 (12)	0.0017 (12)
C3A	0.0511 (19)	0.0459 (16)	0.0351 (14)	−0.0196 (14)	−0.0007 (13)	−0.0026 (13)
C4A	0.0505 (19)	0.0409 (15)	0.0373 (15)	−0.0096 (14)	−0.0049 (13)	−0.0068 (13)
C6A	0.0471 (18)	0.0388 (15)	0.0330 (14)	−0.0123 (13)	0.0014 (12)	0.0036 (12)
C7A	0.071 (3)	0.064 (2)	0.052 (2)	−0.016 (2)	−0.0212 (19)	−0.0103 (18)
O3	0.086 (2)	0.0819 (19)	0.123 (3)	−0.0598 (18)	0.002 (2)	−0.0050 (19)
O4	0.0662 (16)	0.0681 (15)	0.0648 (15)	−0.0399 (12)	−0.0210 (13)	0.0183 (12)
O5	0.0709 (17)	0.0653 (14)	0.0620 (15)	−0.0327 (12)	−0.0126 (13)	0.0160 (12)
N7	0.0485 (17)	0.0447 (15)	0.0663 (19)	−0.0201 (13)	−0.0011 (15)	0.0005 (14)
O6	0.123 (3)	0.078 (2)	0.142 (3)	0.020 (2)	−0.014 (3)	0.011 (2)
O7	0.108 (2)	0.0589 (14)	0.093 (2)	−0.0209 (14)	−0.0085 (19)	0.0116 (14)
O8	0.0704 (18)	0.0586 (15)	0.0870 (19)	−0.0134 (12)	−0.0107 (14)	−0.0056 (13)
N8	0.084 (3)	0.0497 (19)	0.106 (3)	−0.026 (2)	0.000 (3)	−0.002 (2)

### Geometric parameters (Å, °)

**Table:**

Cd1—N1	2.268 (3)	C7—H7C	0.9600
Cd1—N1A	2.274 (2)	O2A—C2A	1.263 (3)
Cd1—O8	2.354 (3)	N1A—C6A	1.333 (3)
Cd1—O4	2.392 (3)	N1A—C2A	1.352 (4)
Cd1—O5	2.479 (3)	N5A—C6A	1.347 (4)
Cd1—O2A	2.542 (3)	N5A—C4A	1.362 (4)
Cd1—O7	2.544 (3)	N5A—H5A	0.8600
Cd1—O2	2.576 (3)	N6A—C6A	1.315 (4)
O2—C2	1.263 (4)	N6A—H6C	0.8600
N1—C6	1.333 (4)	N6A—H6D	0.8600
N1—C2	1.362 (4)	C2A—C3A	1.423 (4)
N5—C6	1.336 (5)	C3A—C4A	1.343 (4)
N5—C4	1.371 (5)	C3A—H3A	0.9300
N5—H5	0.8600	C4A—C7A	1.488 (5)
N6—C6	1.327 (5)	C7A—H7D	0.9600
N6—H6A	0.8600	C7A—H7E	0.9600
N6—H6B	0.8600	C7A—H7F	0.9600
C2—C3	1.408 (5)	O3—N7	1.208 (4)
C3—C4	1.341 (4)	O4—N7	1.251 (4)
C3—H3	0.9300	O5—N7	1.251 (4)
C4—C7	1.489 (6)	O6—N8	1.213 (5)
C7—H7A	0.9600	O7—N8	1.252 (5)
C7—H7B	0.9600	O8—N8	1.239 (5)
			
N1—Cd1—N1A	120.14 (10)	N6—C6—N1	119.3 (4)
N1—Cd1—O8	141.62 (10)	N6—C6—N5	119.5 (3)
N1A—Cd1—O8	87.92 (10)	N1—C6—N5	121.1 (3)
N1—Cd1—O4	88.27 (10)	C4—C7—H7A	109.5
N1A—Cd1—O4	142.19 (9)	C4—C7—H7B	109.5
O8—Cd1—O4	81.88 (11)	H7A—C7—H7B	109.5
N1—Cd1—O5	115.01 (10)	C4—C7—H7C	109.5
N1A—Cd1—O5	91.51 (9)	H7A—C7—H7C	109.5
O8—Cd1—O5	87.40 (11)	H7B—C7—H7C	109.5
O4—Cd1—O5	51.91 (9)	C2A—O2A—Cd1	89.6 (2)
N1—Cd1—O2A	84.62 (9)	C6A—N1A—C2A	120.1 (3)
N1A—Cd1—O2A	54.56 (8)	C6A—N1A—Cd1	140.1 (2)
O8—Cd1—O2A	93.25 (10)	C2A—N1A—Cd1	99.45 (17)
O4—Cd1—O2A	161.62 (8)	C6A—N5A—C4A	121.8 (2)
O5—Cd1—O2A	145.97 (8)	C6A—N5A—H5A	119.1
N1—Cd1—O7	90.67 (12)	C4A—N5A—H5A	119.1
N1A—Cd1—O7	122.16 (11)	C6A—N6A—H6C	120.0
O8—Cd1—O7	51.02 (12)	C6A—N6A—H6D	120.0
O4—Cd1—O7	77.85 (11)	H6C—N6A—H6D	120.0
O5—Cd1—O7	119.94 (12)	O2A—C2A—N1A	116.2 (3)
O2A—Cd1—O7	85.30 (11)	O2A—C2A—C3A	124.2 (3)
N1—Cd1—O2	54.13 (8)	N1A—C2A—C3A	119.6 (3)
N1A—Cd1—O2	85.12 (8)	C4A—C3A—C2A	119.0 (3)
O8—Cd1—O2	163.01 (10)	C4A—C3A—H3A	120.5
O4—Cd1—O2	94.21 (10)	C2A—C3A—H3A	120.5
O5—Cd1—O2	77.32 (10)	C3A—C4A—N5A	119.0 (3)
O2A—Cd1—O2	95.29 (9)	C3A—C4A—C7A	123.9 (4)
O7—Cd1—O2	144.40 (11)	N5A—C4A—C7A	117.0 (3)
C2—O2—Cd1	89.2 (2)	N6A—C6A—N1A	120.3 (3)
C6—N1—C2	119.6 (3)	N6A—C6A—N5A	119.2 (3)
C6—N1—Cd1	139.4 (3)	N1A—C6A—N5A	120.5 (3)
C2—N1—Cd1	100.62 (19)	C4A—C7A—H7D	109.5
C6—N5—C4	121.7 (3)	C4A—C7A—H7E	109.5
C6—N5—H5	119.2	H7D—C7A—H7E	109.5
C4—N5—H5	119.2	C4A—C7A—H7F	109.5
C6—N6—H6A	120.0	H7D—C7A—H7F	109.5
C6—N6—H6B	120.0	H7E—C7A—H7F	109.5
H6A—N6—H6B	120.0	N7—O4—Cd1	97.6 (2)
O2—C2—N1	115.6 (3)	N7—O5—Cd1	93.4 (2)
O2—C2—C3	125.2 (3)	O3—N7—O5	122.1 (3)
N1—C2—C3	119.2 (3)	O3—N7—O4	120.9 (3)
C4—C3—C2	120.1 (4)	O5—N7—O4	117.0 (3)
C4—C3—H3	120.0	N8—O7—Cd1	90.7 (2)
C2—C3—H3	120.0	N8—O8—Cd1	100.3 (3)
C3—C4—N5	118.3 (4)	O6—N8—O7	123.2 (5)
C3—C4—C7	125.1 (4)	O6—N8—O8	120.4 (5)
N5—C4—C7	116.6 (3)	O7—N8—O8	116.4 (4)
			
N1—Cd1—O2—C2	4.3 (2)	O2A—Cd1—N1A—C2A	−2.61 (17)
N1A—Cd1—O2—C2	−128.8 (2)	O7—Cd1—N1A—C2A	−58.1 (2)
O8—Cd1—O2—C2	165.0 (3)	O2—Cd1—N1A—C2A	97.42 (19)
O4—Cd1—O2—C2	89.1 (2)	Cd1—O2A—C2A—N1A	−4.2 (3)
O5—Cd1—O2—C2	138.5 (2)	Cd1—O2A—C2A—C3A	176.0 (3)
O2A—Cd1—O2—C2	−75.1 (2)	C6A—N1A—C2A—O2A	179.1 (3)
O7—Cd1—O2—C2	14.2 (3)	Cd1—N1A—C2A—O2A	4.8 (3)
N1A—Cd1—N1—C6	−119.0 (3)	C6A—N1A—C2A—C3A	−1.1 (5)
O8—Cd1—N1—C6	12.7 (4)	Cd1—N1A—C2A—C3A	−175.4 (2)
O4—Cd1—N1—C6	87.3 (4)	O2A—C2A—C3A—C4A	−178.1 (3)
O5—Cd1—N1—C6	133.3 (3)	N1A—C2A—C3A—C4A	2.1 (5)
O2A—Cd1—N1—C6	−75.7 (4)	C2A—C3A—C4A—N5A	−1.6 (5)
O7—Cd1—N1—C6	9.5 (4)	C2A—C3A—C4A—C7A	178.4 (3)
O2—Cd1—N1—C6	−176.2 (4)	C6A—N5A—C4A—C3A	0.2 (5)
N1A—Cd1—N1—C2	53.2 (2)	C6A—N5A—C4A—C7A	−179.8 (3)
O8—Cd1—N1—C2	−175.10 (19)	C2A—N1A—C6A—N6A	−179.9 (3)
O4—Cd1—N1—C2	−100.5 (2)	Cd1—N1A—C6A—N6A	−8.7 (5)
O5—Cd1—N1—C2	−54.6 (2)	C2A—N1A—C6A—N5A	−0.4 (5)
O2A—Cd1—N1—C2	96.4 (2)	Cd1—N1A—C6A—N5A	170.9 (2)
O7—Cd1—N1—C2	−178.3 (2)	C4A—N5A—C6A—N6A	−179.6 (3)
O2—Cd1—N1—C2	−4.06 (19)	C4A—N5A—C6A—N1A	0.9 (5)
Cd1—O2—C2—N1	−6.4 (3)	N1—Cd1—O4—N7	122.3 (2)
Cd1—O2—C2—C3	174.6 (4)	N1A—Cd1—O4—N7	−19.0 (3)
C6—N1—C2—O2	−178.4 (3)	O8—Cd1—O4—N7	−94.9 (2)
Cd1—N1—C2—O2	7.5 (3)	O5—Cd1—O4—N7	−1.8 (2)
C6—N1—C2—C3	0.6 (5)	O2A—Cd1—O4—N7	−170.5 (2)
Cd1—N1—C2—C3	−173.6 (3)	O7—Cd1—O4—N7	−146.6 (3)
O2—C2—C3—C4	178.7 (4)	O2—Cd1—O4—N7	68.5 (2)
N1—C2—C3—C4	−0.1 (6)	N1—Cd1—O5—N7	−64.1 (2)
C2—C3—C4—N5	−0.7 (6)	N1A—Cd1—O5—N7	171.4 (2)
C2—C3—C4—C7	180.0 (4)	O8—Cd1—O5—N7	83.5 (2)
C6—N5—C4—C3	1.1 (6)	O4—Cd1—O5—N7	1.8 (2)
C6—N5—C4—C7	−179.5 (4)	O2A—Cd1—O5—N7	175.47 (18)
C2—N1—C6—N6	−179.4 (3)	O7—Cd1—O5—N7	42.3 (3)
Cd1—N1—C6—N6	−8.3 (6)	O2—Cd1—O5—N7	−104.0 (2)
C2—N1—C6—N5	−0.2 (5)	Cd1—O5—N7—O3	176.9 (4)
Cd1—N1—C6—N5	171.0 (3)	Cd1—O5—N7—O4	−3.0 (3)
C4—N5—C6—N6	178.6 (3)	Cd1—O4—N7—O3	−176.8 (3)
C4—N5—C6—N1	−0.7 (5)	Cd1—O4—N7—O5	3.2 (4)
N1—Cd1—O2A—C2A	−130.6 (2)	N1—Cd1—O7—N8	170.1 (3)
N1A—Cd1—O2A—C2A	2.76 (18)	N1A—Cd1—O7—N8	−63.0 (3)
O8—Cd1—O2A—C2A	87.9 (2)	O8—Cd1—O7—N8	−7.3 (3)
O4—Cd1—O2A—C2A	161.7 (3)	O4—Cd1—O7—N8	82.0 (3)
O5—Cd1—O2A—C2A	−2.3 (3)	O5—Cd1—O7—N8	50.5 (3)
O7—Cd1—O2A—C2A	138.3 (2)	O2A—Cd1—O7—N8	−105.4 (3)
O2—Cd1—O2A—C2A	−77.41 (19)	O2—Cd1—O7—N8	162.1 (2)
N1—Cd1—N1A—C6A	−118.0 (3)	N1—Cd1—O8—N8	3.3 (4)
O8—Cd1—N1A—C6A	89.6 (3)	N1A—Cd1—O8—N8	143.1 (3)
O4—Cd1—N1A—C6A	15.7 (4)	O4—Cd1—O8—N8	−73.4 (3)
O5—Cd1—N1A—C6A	2.3 (3)	O5—Cd1—O8—N8	−125.3 (3)
O2A—Cd1—N1A—C6A	−174.9 (4)	O2A—Cd1—O8—N8	88.8 (3)
O7—Cd1—N1A—C6A	129.6 (3)	O7—Cd1—O8—N8	7.5 (3)
O2—Cd1—N1A—C6A	−74.9 (3)	O2—Cd1—O8—N8	−151.1 (3)
N1—Cd1—N1A—C2A	54.3 (2)	Cd1—O7—N8—O6	−165.2 (5)
O8—Cd1—N1A—C2A	−98.1 (2)	Cd1—O7—N8—O8	12.1 (4)
O4—Cd1—N1A—C2A	−171.99 (18)	Cd1—O8—N8—O6	164.1 (4)
O5—Cd1—N1A—C2A	174.6 (2)	Cd1—O8—N8—O7	−13.3 (5)

### Hydrogen-bond geometry (Å, °)

**Table:**

*D*—H···*A*	*D*—H	H···*A*	*D*···*A*	*D*—H···*A*
N5—H5···O2A^i^	0.86	1.97	2.779 (3)	156
N6—H6A···O7	0.86	2.26	3.090 (6)	163
N6—H6B···O4^ii^	0.86	2.34	2.892 (4)	122
N6—H6B···O2A^i^	0.86	2.56	3.230 (5)	135
N5A—H5A···O2^iii^	0.86	2.17	2.935 (3)	149
N6A—H6C···O5	0.86	2.36	3.169 (4)	157
N6A—H6D···O2^iii^	0.86	2.25	2.996 (4)	145
N6A—H6D···O3^iv^	0.86	2.26	2.765 (4)	117
C3—H3···O6^v^	0.93	2.31	3.160 (5)	152
C3A—H3A···O8^vi^	0.93	2.42	3.301 (5)	157

Symmetry codes: (i) −*x*+1, −*y*+1, −*z*; (ii) −*x*, −*y*+1, −*z*; (iii) −*x*+1, −*y*, −*z*+1; (iv) −*x*, −*y*, −*z*+1; (v) *x*+1, *y*−1, *z*; (vi) *x*+1, *y*, *z*.
